# Shear bond strength of ceramic fused to CAD-CAM milled alloys

**DOI:** 10.4317/jced.54487

**Published:** 2018-01-01

**Authors:** Niwut Juntavee, San-Eak Oeng

**Affiliations:** 1 Department of Prosthodontics, Faculty of Dentistry, Khon Kaen University, Khon Kaen, Thailand; 2 Division of Biomaterials and Prosthodontics, Faculty of Dentistry, Khon Kaen University, Khon Kaen, Thailand

## Abstract

**Background:**

This study evaluated the metal ceramic bond strength of cast Ni-Cr, cast Co-Cr, sintered Co-Cr and milled Co-Cr alloys to ceramic through two application procedures including the ceramic layering technique and ceramic pressed-on technique.

**Material and Methods:**

Ceramic materials (Ø 8 mm, 1.5 mm thickness) were veneered by either the layering or pressed-on technique to cast Ni-Cr, cast Co-Cr, sintered Co-Cr and hard milled Co-Cr alloy disc (12 × 12 × 0.5 mm) (n=15). All specimens were treated with a thermal cycle process for 500 cycles at the temperature between 5 °C and 55 °C with immerse time of 30 seconds and 5 seconds for specimen transfer. The shear bond strength was determined on a universal testing machine at a crosshead speed of 0.5 mm/min. The de-bonding surfaces were examined under visual inspection and SEM. The metal ceramic interface of specimens for each group was examined in SEM and EDS. The means of bond strength were compared using two-way ANOVA followed by post-hoc Tukey HSD multiple comparison test to determine for statistically significant difference at 95% level of confidence. The Weibull analysis was used for determination survival probability of shear bond strength.

**Results:**

The bond strength of ceramic to sintered Co-Cr alloys was higher than that to others metal alloys. The metal-ceramic mean bond strength was significantly higher for the ceramic pressed-on technique than that of the ceramic layering technique for all tested alloys (*p*<0.05). Weibull analysis of the shear bond strength indicated that the sintered Co-Cr alloys veneered with heat pressed ceramic provided the highest characteristic strength of metal ceramic bond.

**Conclusions:**

The sintered Co-Cr alloys significantly contributed the appropriate bond strength for metal ceramic. Ceramic pressed-on was a reliable technique to enhance bond strength for fabrication the metal ceramic restoration.

** Key words:**Bond strength, sintered alloy, milled alloy, Co-Cr alloys, metal ceramic.

## Introduction

Metal ceramic restorations have been successfully serving as the restorations of choice in dentistry due to the aesthetic property of ceramic veneering over the durable metal substructure (1). The predominated base metal alloys are often attractive for clinician selection for using as a metal substructure due to its lower cost compared to other alloy materials (2-4). The advantages of the mechanical properties, biocompatible, higher hardness, and Young’s modulus of predominated base metal alloy have been broadly indicated for the fabrication of substructure for ceramic veneering in fixed prosthodontics and implant dentistry (5).

With digital dentistry, it is possible to fabricate the metal substructure for metal ceramic restorations by the advancements in additive or subtractive technique from computer-aided design and computer-aided manufacturing (CAD-CAM) (6,7). These techniques require processing metal substructure at the milling center for a very high cost. The introduction of the chalky-like pre-sintered alloy powder blank has offered an advantage to mill substructure in the green stage of powder pre-sintered blank and then later sintered to achieve the final substructure. This process reduces the time and cost in the fabrication of metal substructure. It has been proven that there were comparable mechanical properties of sintered alloy to the hard milling alloy (8). Nevertheless, prosthetic complications upon delamination of the porcelain are crucial for the long-term prognosis of metal ceramic restoration. Several studies have been reported on the survival and complication rates of metal ceramic restorations with porcelain fracture failure ranges from 2-4% upon long-term clinical study (2,3). The systematic review showed that a 10-year risk for fracture of the veneered ceramic from the metal framework was 3.2% (4). Previous studies reported that the sintered alloy exhibited shear bond strength to ceramic comparable to that of the cast and laser sintered alloy upon ceramic application by the layering technique (9).

At present, ceramic can be fused to the metal by two techniques including the ceramic layering technique and the ceramic pressed-on technique. The ceramic layering technique is a conventional ceramic application to construct ceramo-metal restorations. This method requires skills and multiple ceramic applications and firings to achieve final restoration that may introduce more inaccuracy in fabrication (10). The ceramic pressed-on technique requires a press-able glass ceramic to be melted and pressed onto the metal substructure (11). The main advantage of this technique is avoiding multiple ceramic applications and firings that results in achieving precision. This technique also facilitates the processing and provides better distribution of the crystalline phase within the glass matrix (11-13). Moreover, the shrinkage of ceramic upon pressed-on technique is minimized, ensuring a better marginal fit of the restoration (14).

A durable bond at the interface of ceramic and metal substructure plays an essential role for long-term performance in function and aesthetic of metal ceramic restorations (15,16). The metal to ceramic bond is achieved through the chemical bond and micromechanical interlocking to create the adhesion between ceramic and metal (17). The chemical bond between ceramic and metal alloys is derived from the metal oxide on the metal ceramic interface, while the mechanical interlocking is obtained from the nature of the metal surface architecture or the prepared metal surface upon sandblasting or grinding (18-20).

Several studies have investigated the bond strength of the ceramic to metal in ceramo-metal restorations. There was no difference in the bond strength between the conventional ceramic layering technique and ceramic pressed-on technique for high noble metal alloy (10,21). On the contrary, the study on nickel-chromium (Ni-Cr) substructure resulted that the bond strength to pressed-on ceramic was higher than that to conventional layering ceramic (22,23). Nevertheless, there was no information on the shear bond strengths of sintered Co-Cr alloy to ceramic based on ceramic application techniques. This study aimed to evaluate the shear bond strengths of ceramic to metal substructure fabricated from the cast, milled and sintered technique that were veneered with different ceramic application techniques, including conventional ceramic layering and ceramic pressed-on technique.

## Material and Methods

The ceramics, alloys, and their chemical compositions listed in  Table 1 were used for specimen preparation as follow.

**Table 1:**
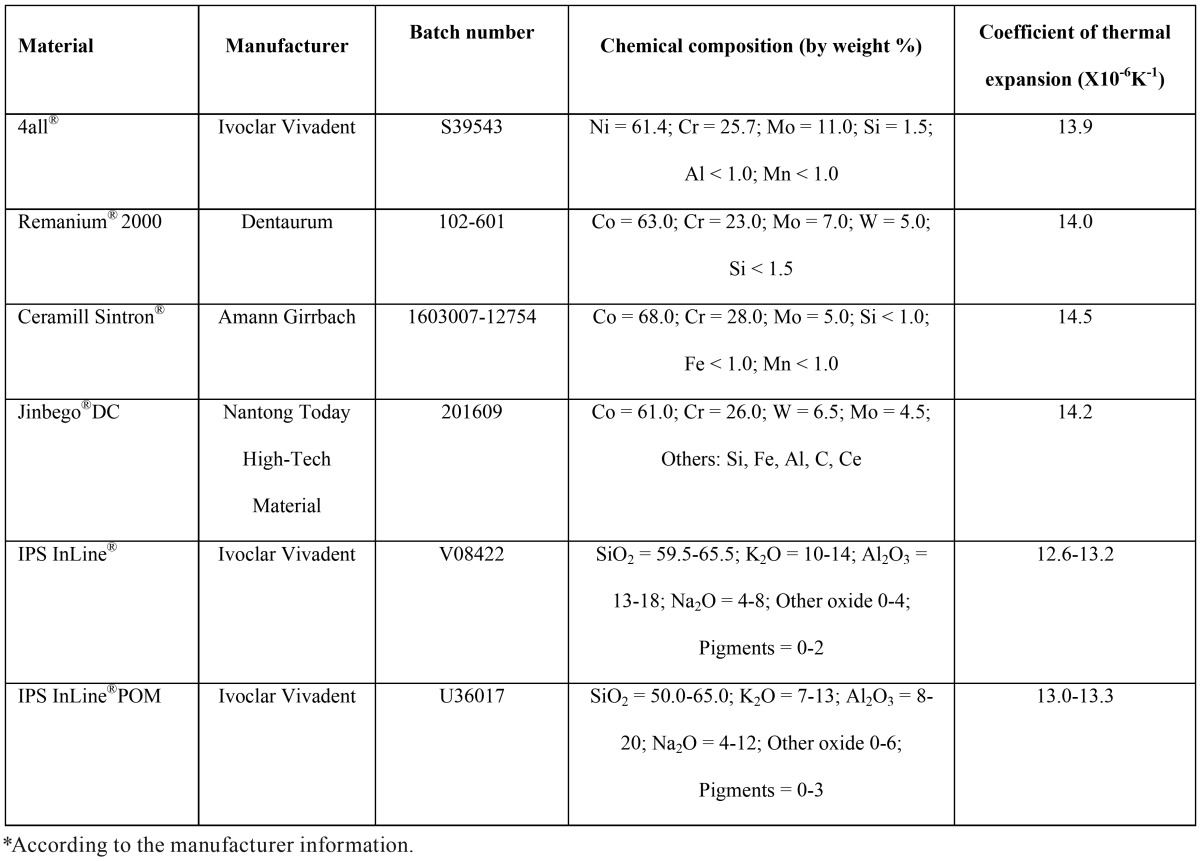
Materials used in this study, chemical composition and properties*.

-Specimen preparation for conventional casting alloy 

Sixty plastic sheets (Coping Material, Keystones, Gibbstown, NJ, USA) in dimensions of 12 × 12 × 0.5 mm were prepared, sprued and invested in the casting rings using phosphate bonded investment (Formula®1, Whip Mix, Louisville, KY, USA). The investments were burnt-out and casted with either Ni-Cr alloy (4all®, Ivoclar Vivadent, Schann, Liechtenstein) to fabricate samples for the cast Ni-Cr (CNi) group or Co-Cr alloy (Remanium® 2000, Dentaurum, Antwerpen, Belgium) to fabricate samples for the cast Co-Cr (CCo) group by using an electric centrifugal casting machine (Fornax®T, Bego, Bremen, Germany). There were thirty samples for each group. The cast metal samples were divested and sandblasted with 110 microns aluminum oxides abrasive (Korox®110, Bego, Bremen, Germany) to remove remaining residue.

-Specimen preparation for sintered alloy 

A Co-Cr pre-sintered powder alloy blank (Ceramill Sintron®, Amann Girrbach, Koblach, Austria) was prepared into the square shape of dimension 14 × 14 × 0.7 mm using a precision cutting machine (Megatrome®T180, Presi, Eybens, France). The samples were fully sintered in a furnace (Ceramill® Argotherm, Amann Girrbach, Koblach, Austria) under argon gas at 1300 °C for six hours. Upon metal sintering process, the sintering shrinkage of metal for an approximately 15% was derived and resulted in a final dimension of metal sample to be 12 × 12 × 0.5 mm for this sintered metal (SMCo) group.

-Specimen preparation for hard milled alloy 

Thirty samples of Co-Cr alloy disc specimens, of dimension 12 × 12 × 0.5 mm, were prepared from the hard Co-Cr alloys blank (Jinbego®DC, Nantong Today High-Tech Material, Jiansu, China) through a precision cutting machine to fabricate alloy samples for the milled Co-Cr (MCo) group.

-Surface preparation of alloy 

The surface of the alloy specimens was grinded with stone bur (Coral stones®, Shofu, Kyoto, Japan) in one direction at a speed of 20,000 rpm/min and subsequently sandblasted with 110 micron aluminum oxide (Korox®110, Bego, Bremen, Germany) at 2 bar pressure for 10s using sandblaster (Basic®eco, Renfert, Helzinger, German) with the tip set at a 10 mm distance from the metal surface. The specimens were ultrasonically cleaned in the distilled water for 30 minutes and steam cleaned for 15 seconds. The cast Ni-Cr group was heat treated in a ceramic furnace (Progammat®P100, Ivoclar Vivadent, Schann, Liechtenstein) according to the manufacturer’s recommendation.

-Opaque ceramic application

The paste opaque ceramic shade A3 (IPS InLine®, Ivoclar Vivadent, Schann, Liechtenstein) was applied to the middle of the metal specimens and fired two times according to the manufacturer’s firing schedule in order to derive for the opaque dimension of 8 mm in diameter and 0.1 mm thickness. The first opaque layer was thinly applied and fired. The second opaque layer was entirely applied covering the previous opaque layer and fired to derive for silky-mat shiny surface.

-Conventional ceramic layering technique

Fifteen samples from each alloy group were veneered with ceramic using the conventional layering technique (L). A creamy mix consistency of body ceramic shade A3 (IPS InLine®, Ivoclar Vivadent, Schann, Liechtenstein) was applied on the opaque surface and condensed on an ultrasonic ceramic condenser (Ceramosonic®, Unitek, Osaka, Japan). The specimens were fired in the ceramic furnace according to the manufacturer’s instructions. The ceramic application technique was allowed for two times in order to reach the dimension of ceramic specimens of diameter 8 mm and 1.5 mm thickness, and then glazed.

-Ceramic pressed-on veneering technique

Fifteen specimens from each metal alloy group were coated with casting wax (Blue inlay casting wax®, Kerr, Charlotte, NC, USA) with the diameter of 8mm and thickness of 1.5 mm to fabricate a wax pattern on the opaque layer of the specimens. The specimens were sprued to the wax portion and invested using a proprietary investment material (IPS PressVEST Speed®; Ivoclar Vivadent, Schann, Liechtenstein) and burnt-out in the furnace (Kavo-EWL®5645, Kavo, Warthausen, Germany). After completion of the burn out process, the rings were transferred to a ceramic pressing furnace (Programat®EP5000, Ivoclar Vivadent, Schann, Liechtenstein) for ceramic pressing procedure (P) using ceramic ingots shade A3 (IPS InLine®POM, Ivoclar Vivadent, Schann, Liechtenstein). Once the pressing process was finished, the investments were divested. The samples were polished, finished, and then glazed.

-Thermal cycle technique

All specimens were subjected to a thermal cycling process for 500 cycles at the temperature between 5°C and 55°C for 30 seconds immersion in each temperature bath and 5 seconds for specimen transfer between each bath in order to assess for the durability of metal ceramic bond strength.

-Evaluation of shear bond strength

The shear bond strength test was performed by subjecting the ceramic-metal junction to a compression shear apparatus on a universal testing machine (Lloyd®LR 30k, Lloyd, Leicester, England) at crosshead speed of 0.5 mm/min (24,25). The loads at failure of ceramic bonded to metal alloy were recorded. The shear bond strength (σ) was calculated by dividing the failure load (P) by the area of interface between ceramic to metal (A) as equation 1 σ= P/A ……………….Equation 1.

In which: σ is shear bond strength (MPa), P is maximum load (newton) required for producing fracture, and A is the adhesive cross-sectional bonding area in mm2 (where A = ¶r2). The r denotes for radius of the bonded area.

-Microscopic examination

The de-bonding surfaces were examined visually under optical stereomicroscope (Nikon, Melville, NY, USA) and photomicrograph upon scanning electron microscopy (S-3000N, Hitachi, Tokyo, Japan) for characterizing the type of bonding failure (cohesive failure in metal, adhesion failure at ceramic-metal interface, cohesion failure in ceramic, and mixed type of failure). The metal-ceramic interface in each group was examined with the scanning electron microscope and energy dispersive x-ray spectroscopy (EDS, Oxford instrument, Oxfordshire, United Kingdom).

-Statistical Analysis of data

The data were statistically analyzed using statistical software (SPSS/PC Version 20, IBM, Armonk, NY, USA). An analysis of variance (ANOVA) was used to determine the statistically significant difference of shear bond strength upon the alloys used as well as ceramic veneering techniques. Post-hoc Tukey HSD multiple comparison was used to determine the difference between such factors at 95% level of confidence. Weibull analysis was performed to determine for reliability of bond strength using Weibull statistics (Weibull++® (ReliaSoft, Tucson, AZ, USA).

## Results

**Figure 1:**
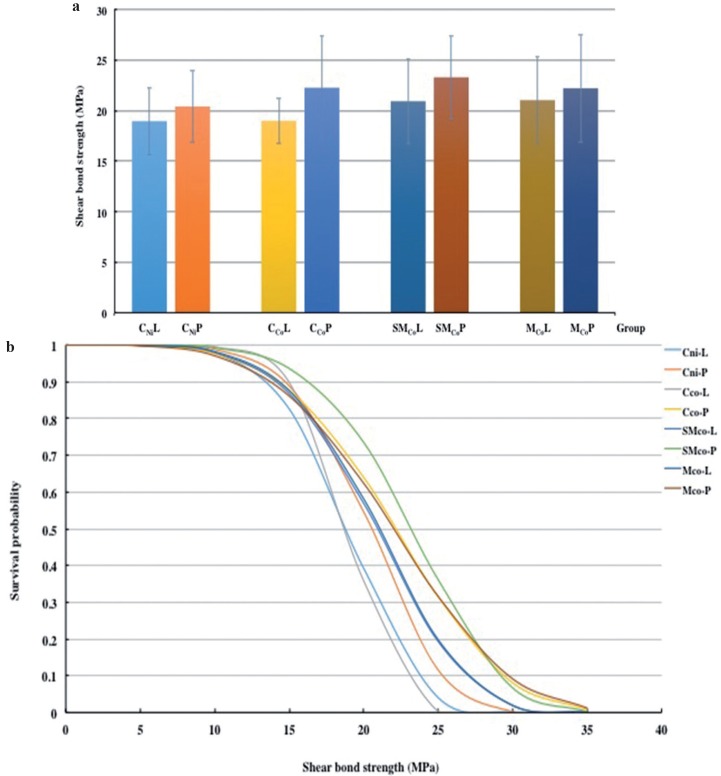
(a) Bar chart representing the comparison of shear bond strength, and (b) line chart representing the comparison of Weibull survival probability of shear bond strength for ceramic veneered to cast Ni-Cr (CNi), casting Co-Cr (CCo), sintered Co-Cr (SMCo) and milled Co-Cr (MCo) with either ceramic layering (L) or ceramic pressed-on technique (P).

The mean, standard deviation, 95% confidence interval, Weibull’s modulus, and characteristic strength for metal-ceramic shear bond strength for each group were presented in t Table 2 and figure 1(a). Upon the ceramic application by layering technique (L), the highest bond strength was demonstrated in the group MCo-L (21.03±4.28 MPa), and followed by SMCo-L (20. 92±4.20 MPa), CCo-L (18.98±2.26 MPa), and CNi-L (18.95±3.32 MPa). For the group of pressed-on ceramic (P), the highest shear bond strength was indicated in the group SMCo-P (23. 29±4.10 MPa), and followed by CCo-P (22.26±5.12 MPa), MCo-P (22.20±5.53 MPa), and CNi-P (20.40±3.58 MPa). The mean bond strength of the heat pressed ceramic technique (P) was higher than that of the ceramic layering technique (L) for all alloy groups. Two-way ANOVA showed a statistically significant difference upon the ceramic veneering technique (*p*<0.05), but no significant difference upon metal tested p>0.05) and interaction between factors (*p*>0.05) as shown in  Table 3 Weibull analysis for characteristic strength of shear bond indicated relative survival probability of bond strength to be ranked from the highest to lowest as SMCo-P (24.92 MPa), CCo-P (24.17 MPa), MCo-P (24.31 MPa), MCo-L (22.66 MPa), SMCo-L (22.54 MPa), CNi-P (21.84 MPa), CNi-L (20.24 MPa), and CCo-L (19.92) groups (Fig. 1b).

**Table 2:**
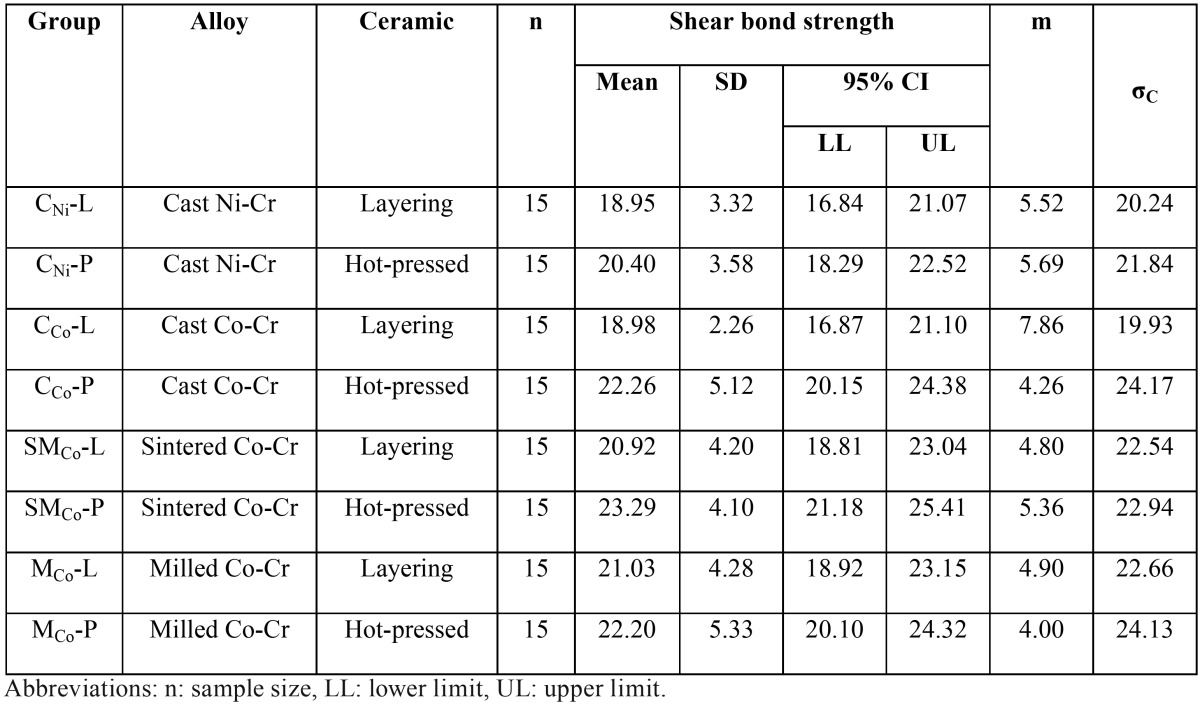
Mean, standard deviation (SD), 95% confidential interval (CI), Weibull’s modulus (m), and characteristic strength (σC) for shear bond strength (MPa).

**Table 3:**
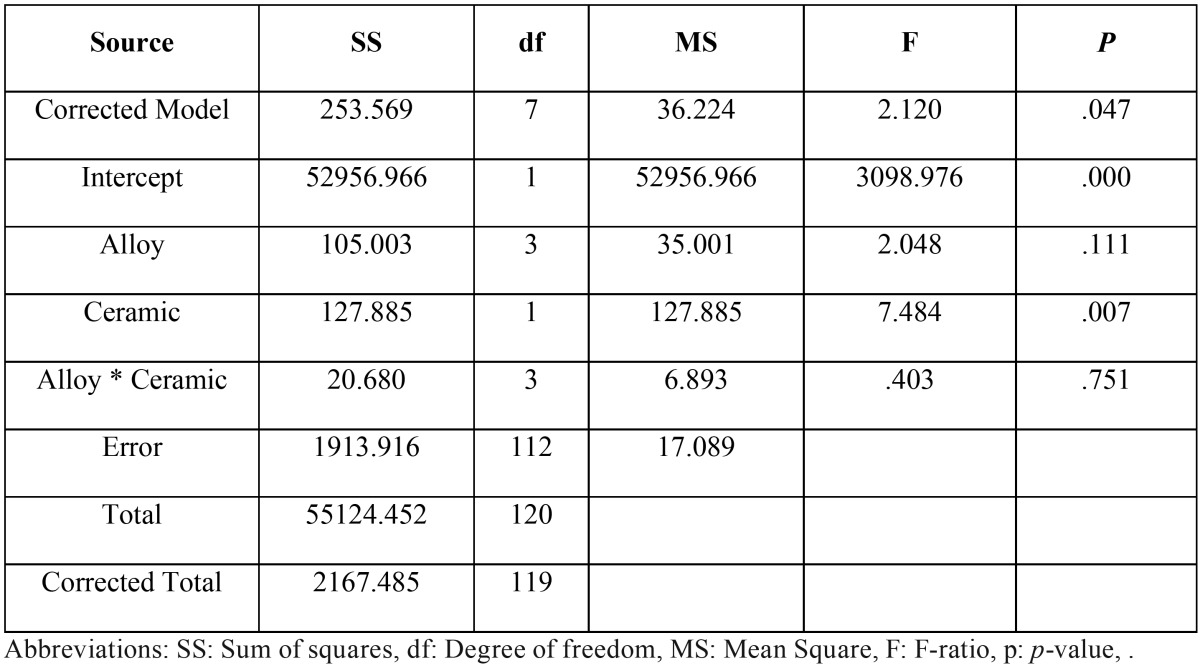
Two-way analysis of variance (ANOVA) of shear bond strength.

**Figure 2:**
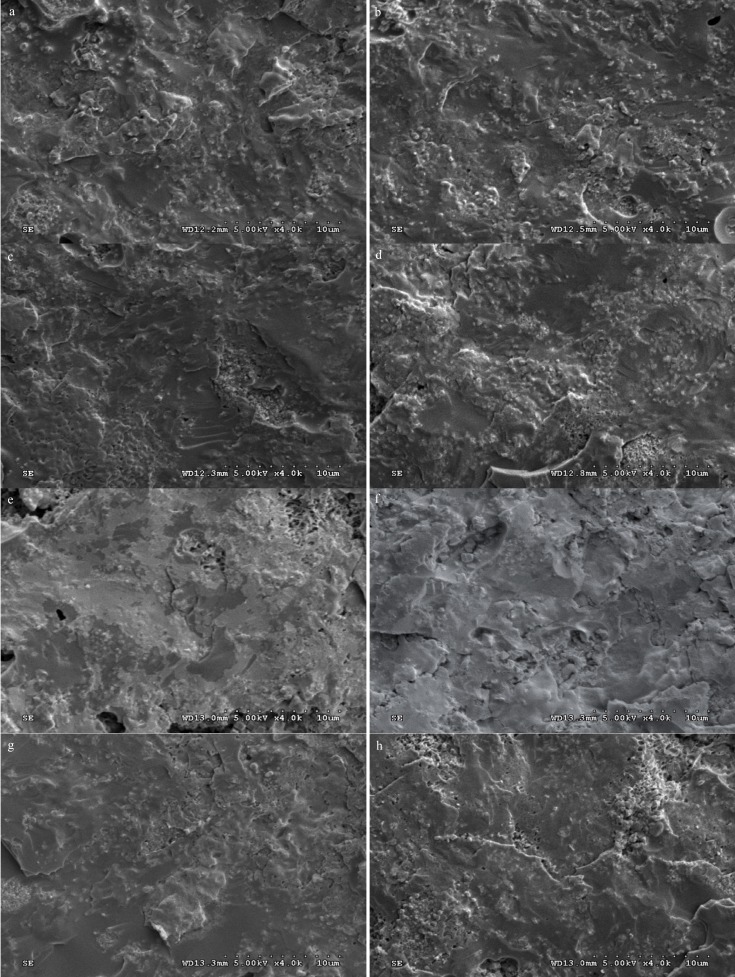
SEM of fracture surface of specimens upon ceramic application by layering technique to (a) cast Ni-Cr, (b) cast Co-Cr, (c) sintered Co-Cr, and (d) milled Co-Cr and by pressed-on technique to (e) cast Ni-Cr, (f) cast Co-Cr, (g) sintered Co-Cr, and (h) milled Co-Cr (at X4000 magnification).

**Figure 3:**
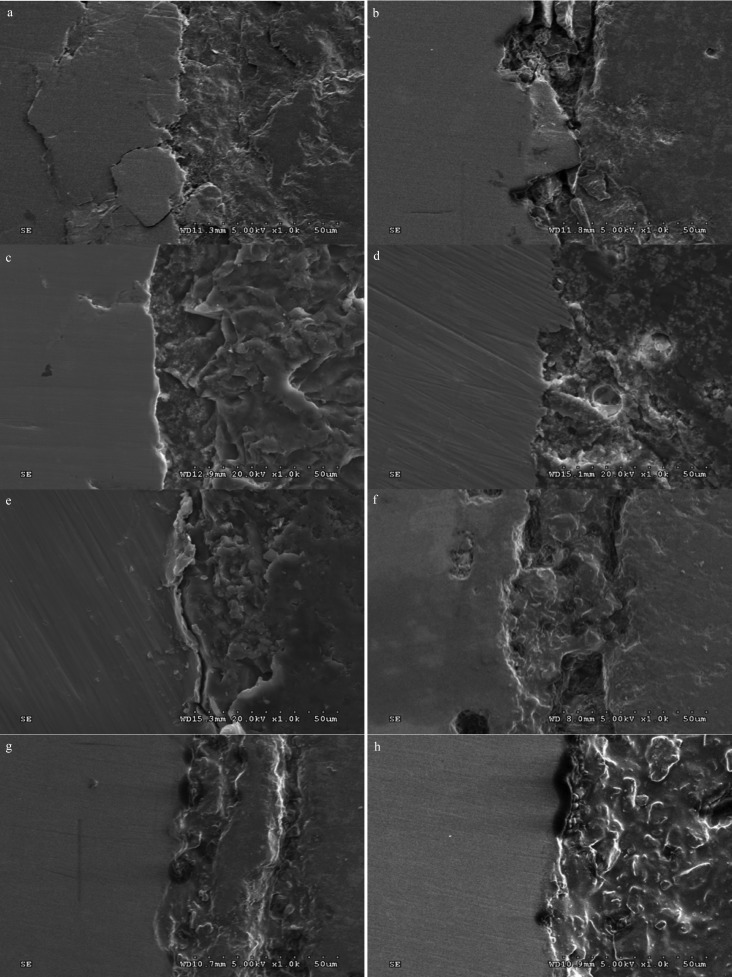
SEM of metal-ceramic interface of specimens upon ceramic application by layering technique to (a) cast Ni-Cr, (b) cast Co-Cr, (c) sintered Co-Cr and (d) milled Co-Cr and by pressed-on technique to (e) cast Ni-Cr, (f) cast Co-Cr, (g) sintered Co-Cr, and (h) milled Co-Cr (at X4000 magnification).

The specimens demonstrated adhesive type of bond failure at the ceramic-alloy interface upon visual and microscopic examination as shown in  Table 4 The scanning electron photomicrograph of the fracture specimen revealed predominantly adhesive metal-ceramic failure at the interfacial adherence zone with minute amount of ceramic particles on the irregularity surface of alloy (Fig. 2). The SEM photomicrograph at the metal ceramic interface exhibited some micro-gaps on the casting alloy groups comparing to the others. The interface for the groups of ceramic pressed-on veneering technique revealed rather more harmonized inter-digitation between metal and ceramic than the groups of the conventional ceramic layering technique (Fig. 3).

**Table 4:**
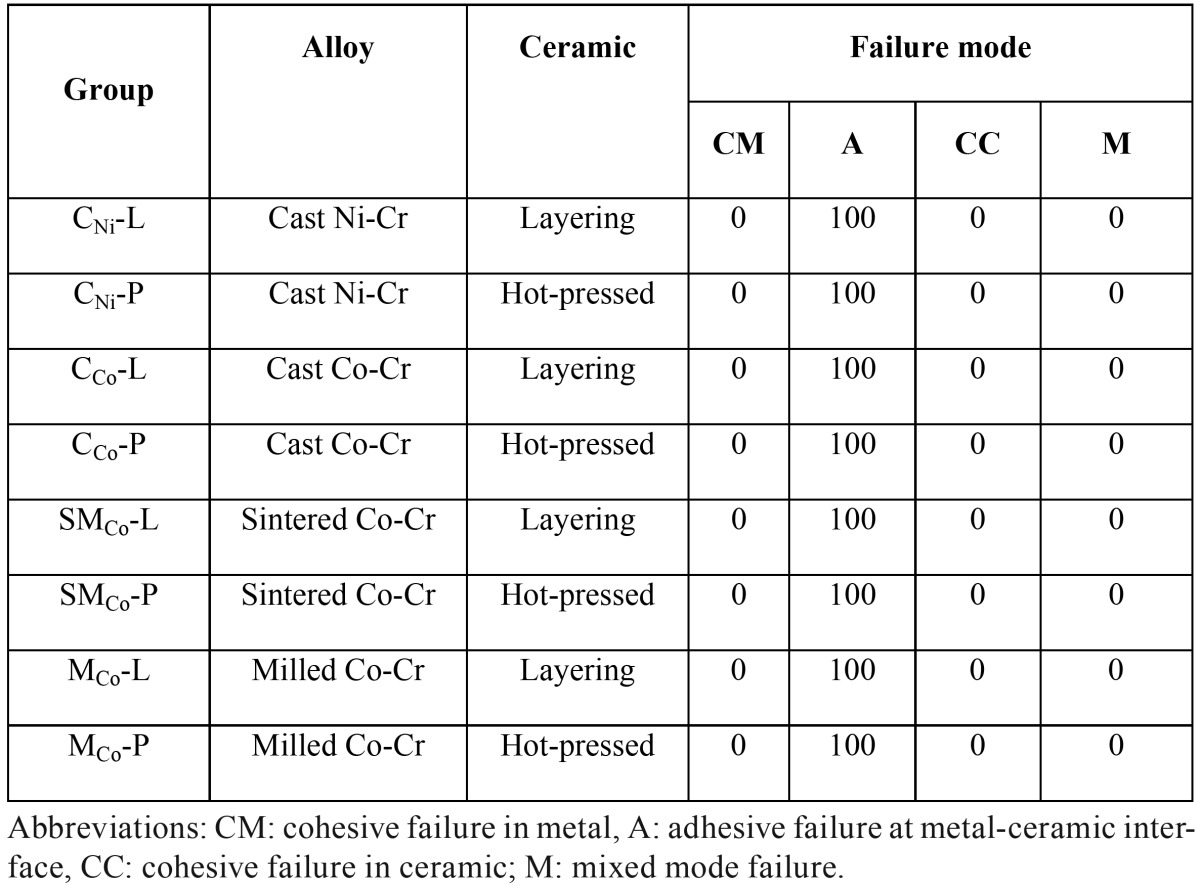
Percentage (%) of failure mode of shear bond strength testing for each group.

## Discussion

On the basis of the results of the present study, the sintered Co-Cr alloy exhibited comparable ceramic bond strength to the conventional cast alloys and hard milled alloy for both ceramic application techniques. This study was supported by other studies that investigated solely the ceramic layering technique on bond strength (9). The ceramic pressed-on veneering technique significantly improved the bond strength of the ceramo-metal restoration. It was consistent with the results of the previous studies (19,22,23).

It is a fact that the bonding of ceramic veneered to metal depends on mechanical interlocking, true chemical bonding and Van der Waals force. The mechanical bond mechanism is the most important that influences the overall metal-ceramic bond strength which depends mostly on the surface architecture of ceramic alloy. The chemical bond is achieved through a metal-ceramic oxide interface. The Van der Waals force influences only a minor contribution to overall bond strength. The surface architecture of ceramic alloy is achieved through the nature of alloy fabrication or may be modified through metal surface preparation. The surface treatment of the metal substructure is important for metal-ceramic bonding (16). Rough surfaces of metal substructure are reported to promote the wetting ability of ceramic better than smooth surfaces, which results in enhancing the metal-ceramic bonding strength (17). Metal surface roughness provides the micromechanical retention that occurs as the ceramic flows onto the irregularity of metal surface, thus enhancing the metal-ceramic adhesion. Furthermore, metal surface roughness can enhance the bond strength by increasing the surface area of the bonding. It was reported that the metal surface treatment by blasting with 110 microns aluminum oxide significantly improved the bond strength of metal-ceramic (19). On the other hand, a rough metal surface may induce the possibility of trapping air pocket and contaminants, which may lead to gas formation during ceramic firing, causing porosity production in the ceramic and may affect the mechanical bond (18). It implies that the conventional ceramic veneering method tends to produce a higher risk of air trap occurrence than the ceramic pressed-on veneering method. This is the reason supporting the presented results that the ceramic pressed-on to metal alloy exhibited higher bond strength than that of the ceramic layering technique.

The chemical bond mechanism occurred through the oxide layer and exhibited an adhesion between ceramic to metal. True chemical bonding results from electron transfer between the oxygen of the glassy phase of ceramic and an oxidized metal surface. It is formed when the ceramic is fired above its glass transition temperature such that it can flow and fuse with the oxides at the metal surface by migration of the oxides into the ceramic (20). The predominated based metal alloy containing Co, Cr and Ni had prominent interactions with ceramics (17). The elements interchange across the metal-ceramic interface strongly provided chemical bonding mechanism.

There are many available methods to measure metal-ceramic bond strength. Among those tests, the result of the shear bond strength test is characterized by not being influenced by the Young’s modulus of the alloy as happens for the bending test (24,25). The circular-planar shear test provides the advantages of controllable standardization of the testing procedure while the specimens were submitted to testing. This type of test is highly reliable because it involved minimal experimental variables and induced the least residual stresses at the metal-ceramic interface. The shear apparatus used in this study is able to induce the shear stress concentration directly to the interface and can assure for precise determination of bond strength.

## Conclusions

Sintered metal alloy for CAD-CAM exhibited shear bond strength to ceramic comparable with cast metal alloys and hard milled alloy either ceramic application by ceramic pressed-on technique or ceramic layering technique. The ceramic pressed-on technique significantly provided higher metal-ceramic bond strength for metal ceramic restorations than that of conventional layering technique. It is suggested that the heat pressed technique is recommended in ceramic application to dental alloy. Sintered alloy is more preferable selection for metal ceramic restoration since it exhibited a suitable survival probability as well as reliable shear bond strength compared to others.
